# 534 Retrospective Study Comparing Acute Healing Outcomes of Two Epidermal Skin Substitutes in Paediatric Partial-Thickness Burns

**DOI:** 10.1093/jbcr/irae036.168

**Published:** 2024-04-17

**Authors:** Ameer Khamise, Hadas Lapid, Ankit Mishra, Alexandra M Murray

**Affiliations:** The University of Buckingham, Aylesbury, England; Afeka College of Engineering, Tel-Aviv, Tel Aviv; Stoke Mandeville Hospital, Aylesbury, England; Buckinghamshire Healthcare NHS Trust, Aylesbury, England; The University of Buckingham, Aylesbury, England; Afeka College of Engineering, Tel-Aviv, Tel Aviv; Stoke Mandeville Hospital, Aylesbury, England; Buckinghamshire Healthcare NHS Trust, Aylesbury, England; The University of Buckingham, Aylesbury, England; Afeka College of Engineering, Tel-Aviv, Tel Aviv; Stoke Mandeville Hospital, Aylesbury, England; Buckinghamshire Healthcare NHS Trust, Aylesbury, England; The University of Buckingham, Aylesbury, England; Afeka College of Engineering, Tel-Aviv, Tel Aviv; Stoke Mandeville Hospital, Aylesbury, England; Buckinghamshire Healthcare NHS Trust, Aylesbury, England

## Abstract

**Introduction:**

Paediatric burns are a significant global health issue; partial thickness (PT) wounds from scalds are a common injury pattern. Skin substitutes, such as collagen based (CB) dressings, are frequently used in managing these cases. We conducted a novel comparative study analysing the acute healing outcomes of a CB dressing and a novel biosynthetic cellulose (BC) dressing in paediatric PT burns.

**Methods:**

All paediatric patients (age < 18) with PT burns that were managed with either the CB or the BC dressings over a four-year period at our paediatric burns centre were included. Data on adherence, infection, length of stay, complete healing time, acute follow-up time, and return to theatre were extracted and a statistical analysis performed.

**Results:**

Of the 89 patients included, 28 received BC dressing and 61 received CB dressing. Mean Total Body Surface Area (TBSA) was 5.9% (range 1-15) and median age was 23 months (range 8-169). BC dressing had a higher adherence rate (85.7% vs 78.7%, P=0.767) and a lower infection rate (3.6% vs 16.4%, P=0.164) than CB dressing. These trends were preserved when superficial PT only and superficial and deep PT sub-groups were analysed. Healing and infection rates were unaffected by post-operative antibiotics in both groups (P=1 and P=0.803, respectively). Return to theatre for further surgery was required in 10.7% of the patients for BC and in 6.6% for CB dressing (P=0.507). Median time to complete healing was longer in BC (19.5 vs 16 days, P=0.279). BC dressing median hospitalisation length was 1 day compared to 2 days for CB dressing (P=0.706).

**Conclusions:**

The findings demonstrate that acute healing outcomes with the BC dressing in paediatric PT burns are comparable to those with the CB dressing, establishing the BC dressing as a viable alternative given its advantages in cost and avoiding porcine components. However, further prospective studies are warranted to compare acute and long-term outcomes with these products.

**Applicability of Research to Practice:**

The study will directly inform clinical decision making on the use of frequently applied epidermal skin substitutes when managing paediatric partial thickness burns.

